# Supplementary material for: Structural basis of Mycobacterium Fluoroquinolone Resistance Protein D (MfpD), a versatile pathogeny protein from the mfp conservon of Mycobacterium tuberculosis
Source: bioRxiv. 2026 Mar 18:2026.03.03.709265. Preprint. [Version 2] doi: 10.64898/2026.03.03.709265 (PMC13015309; doi:10.64898/2026.03.03.709265)
Supplement: Supplement 1 [file NIHPP2026.03.03.709265v2-supplement-1.pdf]

bioRxiv preprint doi: https://doi.org/10.64898/2026.03.03.709265; this version posted March 18, 2026. The copyright holder for this preprint (which was not certified by peer review) is the author/funder, who has granted bioRxiv a license to display the preprint in perpetuity. It is made available under aCC-BY 4.0 International license.

Supplementary Information

Structural basis of *Mycobacterium* Fluoroquinolone Resistance Protein D (MfpD), a versatile pathogeny protein from the *mfp* conservon of *Mycobacterium tuberculosis*

Antoine Gedeon[1#], Maureen Micaletto[1,2], Daniela Megrian[3], Elodie Carmen Leroy[1,4], Elorri Barbier[1], Bertrand Raynal[5], Ahmed Haouz[6], Pedro M. Alzari[1], Claudine Mayer[1,8,9] Stéphanie Petrella[1,7,9*]

[1]Institut Pasteur, Université Paris Cité, Unité de Microbiologie Structurale, CNRS UMR 3528, 75015 Paris, France

[2]Living Systems Institute, University of Exeter, United Kingdom; Biosciences, University of Exeter, United Kingdom

[3]Institut Pasteur de Montevideo, Bioinformatics Unit, 11200 Montevideo, Uruguay

[4]Fondazione Human Technopole, 20157 Milan, Italy

[5]Institut Pasteur, Université Paris Cité, Plateforme de Biophysique Moléculaire, C2RT, CNRS UMR 3528, 75015 Paris, France

[6]Institut Pasteur, Université Paris Cité, Plateforme de Cristallographie, C2RT, CNRS UMR 3528, 75015 Paris, France

[7]Institut Pasteur, Université Paris Cité, Bacterial Cell Cycle Mechanisms Unit, 75015 Paris, France

[8]Complex Systems and Translational Bioinformatics (CSTB), ICube Laboratory, UMR7357, University of Strasbourg, 1 rue Eugène Boeckel, 67000 Strasbourg, France.

[9]Faculté des Sciences, Université Paris Cité, UFR Sciences du Vivant, 75013 Paris, France

[#]Current address: Sorbonne Université, Institut de Biologie Paris-Seine (IBPS), CNRS UMR8263, INSERM U1345, Unité Développement, Adaptation et Vieillissement (Dev2A), Équipe RNAModBio, 75005 Paris, France

*Corresponding author: stephanie.petrella@pasteur.fr

bioRxiv preprint doi: https://doi.org/10.64898/2026.03.03.709265; this version posted March 18, 2026. The copyright holder for this preprint (which was not certified by peer review) is the author/funder, who has granted bioRxiv a license to display the preprint in perpetuity. It is made available under aCC-BY 4.0 International license.

Supplementary Table 1. Identity percentages for Mfp proteins between M. tuberculosis H37Rv and M. smegmatis ATCC 700084. Molecular weights are listed for reference.

	M. tuberculosis	***M. smegmatis***	***%Identity***
MfpA	Rv3361c<br>(20k Da)	MSMEG_1641<br>(21 kDa)	66%
MfpB	Rv3362c<br>(20 kDa)	MSMEG_1640<br>(21 kDa)	78%
MfpC	Rv3363c<br>(13 kDa)	MSMEG_1639<br>(13 kDa)	68%
MfpD	Rv3364c<br>(13 kDa)	MSMEG_1638<br>(14 kDa)	83%
MfpE	Rv3365c<br>(93 kDa)	MSMEG_1637<br>(97 kDa)	55%

bioRxiv preprint doi: https://doi.org/10.64898/2026.03.03.709265; this version posted March 18, 2026. The copyright holder for this preprint (which was not certified by peer review) is the author/funder, who has granted bioRxiv a license to display the preprint in perpetuity. It is made available under aCC-BY 4.0 International license.

Supplementary Table 2. Hydrogen bonds implicated in MfpD dimerisation. Interactions are detected according to a PISA analysis . Note that no disulfide bonds, covalent bonds, or salt bridges exist.

	Structure 1		***Distance (Å)***	***Structure 2***	
1	B:Arg	47 (NH1)	3.01	A:Leu	68 (O)
2	B:Gln	73 (N)	2.92	A:Gln	82 (OE1)
3	B:Leu	75 (N)	2.81	A:Glu	80 (O)
4	B:Gln	76 (N)	3.35	A:Glu	80 (O)
5	B:Gln	76 (NE2)	2.72	A:Glu	80 (OE1)
6	B:Val	78 (N)	2.99	A:Val	78 (O)
7	B:Glu	80 (N)	2.81	A:Gln	76 (O)
8	B:Gln	82 (N)	3.22	A:Gly	71 (O)
9	B:Gln	82 (N)	2.90	A:Gln	73 (O)
10	B:Asn	83 (N)	2.93	A:Gly	71 (O)
11	B:Asn	83 (ND2)	3.04	A:Asp	70 (O)
12	B:Leu	68 (O)	2.91	A:Arg	47 (NH1)
13	B:Gln	82 (OE1)	3.00	A:Gln	73 (N)
14	B:Glu	80 (O)	2.77	A:Leu	75 (N)
15	B:Glu	80 (O)	3.27	A:Gln	76 (N)
16	B:Val	78 (O)	2.95	A:Val	78 (N)
17	B:Gln	76 (O)	2.82	A:Glu	8O (N)
18	B:Gly	71 (O)	3.32	A:Gln	82 (N)
19	B:Gln	73 (O)	2.91	A:Gln	82 (N)
20	B:Gly	71 (O)	2.80	A:Asn	83 (N)
21	B:Asp	70 (O)	3.03	A:Asn	83 (ND2)

bioRxiv preprint doi: https://doi.org/10.64898/2026.03.03.709265; this version posted March 18, 2026. The copyright holder for this preprint (which was not certified by peer review) is the author/funder, who has granted bioRxiv a license to display the preprint in perpetuity. It is made available under aCC-BY 4.0 International license.

Supplementary Table 3. Hydrogen bonds (entries 1 to 16) and salt bridges (entries 17-18) implicated in Ttm MglB dimerisation. Interactions are detected according to a PISA analysis . Note that no disulfide bonds or covalent bonds exist.

	Structure 1		***Distance (Å)***	***Structure 2***	
1	A:Gln	83 (N)	2.71	A:Gln	88 (O)
2	A:Glu	84 (N)	3.18	A:Gln	88 (O)
3	A:Val	86 (N)	2.89	A:Val	86 (O)
4	A:Gln	88 (N)	2.84	A:Glu	84 (O)
5	A:Glu	90 (N)	3.71	A:Glu	79 (OE1)
6	A:Arg	91 (NH1)	3.10	A:Leu	77 (O)
7	A:Arg	91 (NH2)	2.31	A:Gly	78 (O)
8	A:Met	92 (N)	3.42	A:Glu	79 (OE1)
9	A:Leu	77(O)	3.70	A:Arg	91 (NH1)
10	A:Gly	78 (O)	2.31	A:Arg	91 (NH2)
11	A:Glu	79 (OE1)	3.42	A:Met	92 (N)
12	A:Glu	79 (OE1)	3.71	A:Glu	90 (N)
13	A:Glu	84 (O)	2.84	A:Gln	88 (N)
14	A:Val	86 (O)	2.89	A:Val	86 (N)
15	A:Gln	88 (O)	3.18	A:Glu	84 (N)
16	A:Gln	88 (O)	2.71	A:Gln	83 (N)
17	A:His	87 (NE2)	3.31	A:Glu	85 (OE2)
18	A:Glu	85 (OE2)	3.31	A:His	87 (NE2)

bioRxiv preprint doi: https://doi.org/10.64898/2026.03.03.709265; this version posted March 18, 2026. The copyright holder for this preprint (which was not certified by peer review) is the author/funder, who has granted bioRxiv a license to display the preprint in perpetuity. It is made available under aCC-BY 4.0 International license.

Supplementary Table 4. Confidence metrics for AlphaFold 3 modeled complexes. Interface predicted template modeling (ipTM) and predicted template modeling (pTM) values higher than 0.80 mean that structures represent high-quality predictions. n.a., not applicable.

	ipTM	***pTM***
1x MfpB	n.a.	0.81
1x MfpB – 1x MgGTP	0.95	0.80
1x MfpB – 1x GDP	0.93	0.79
1x MfpB – 2x MfpD - 1xMgGTP	0.89	0.90
1x MfpB – 2x MfpD - 1xGDP	0.79	0.82

bioRxiv preprint doi: https://doi.org/10.64898/2026.03.03.709265; this version posted March 18, 2026. The copyright holder for this preprint (which was not certified by peer review) is the author/funder, who has granted bioRxiv a license to display the preprint in perpetuity. It is made available under aCC-BY 4.0 International license.

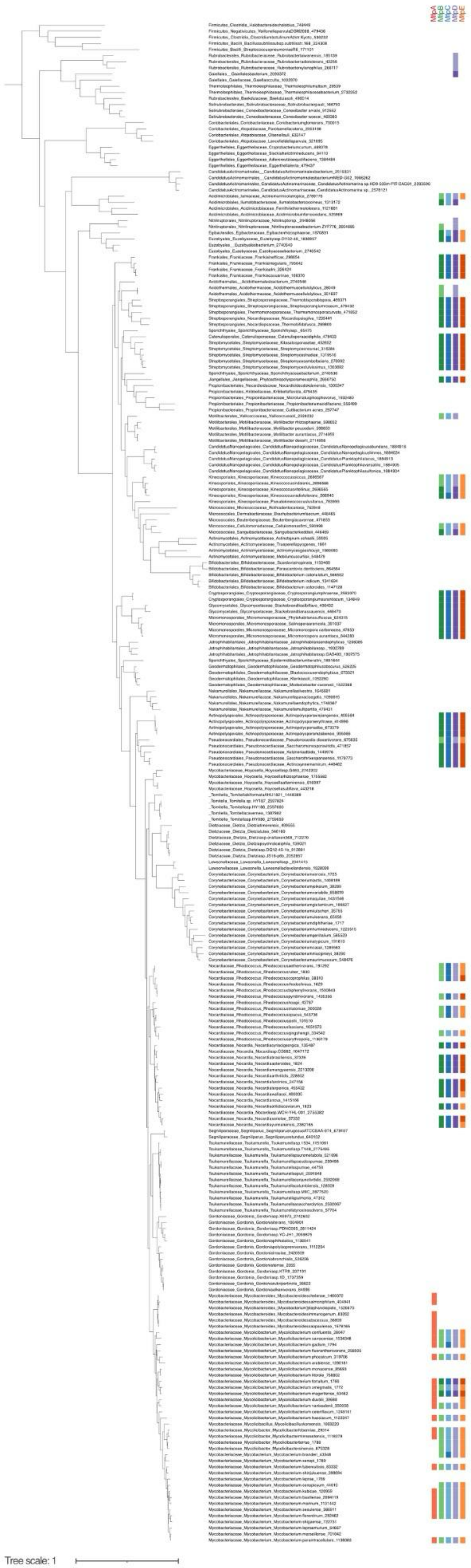

bioRxiv preprint doi: https://doi.org/10.64898/2026.03.03.709265; this version posted March 18, 2026. The copyright holder for this preprint (which was not certified by peer review) is the author/funder, who has granted bioRxiv a license to display the preprint in perpetuity. It is made available under aCC-BY 4.0 International license.

Supplementary Figure 1. Phyletic pattern of the presence of genes mfpA, mfpB, mfpC, mpfD and mfpE mapped onto a reference phylogeny of the Actinobacteria. A colored square indicates the identification of the gene in at least half of the genomes analysed for the corresponding lineage. A darker shade of the color indicates the identification of more than one copy of the gene per genome. The scale bar represents the average number of substitutions per site. For a summarized version, see Figure 1d.

bioRxiv preprint doi: https://doi.org/10.64898/2026.03.03.709265; this version posted March 18, 2026. The copyright holder for this preprint (which was not certified by peer review) is the author/funder, who has granted bioRxiv a license to display the preprint in perpetuity. It is made available under aCC-BY 4.0 International license.

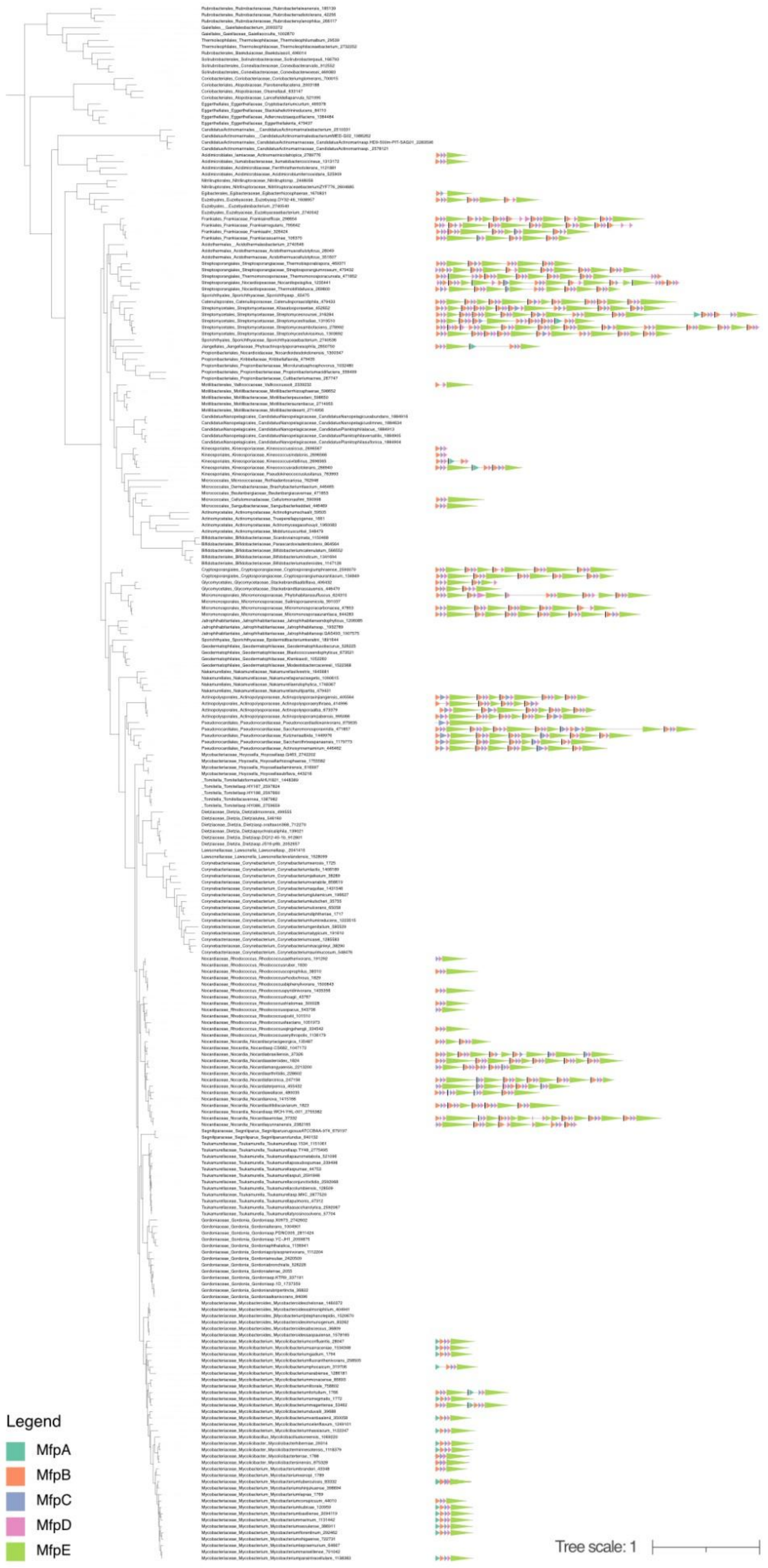

bioRxiv preprint doi: https://doi.org/10.64898/2026.03.03.709265; this version posted March 18, 2026. The copyright holder for this preprint (which was not certified by peer review) is the author/funder, who has granted bioRxiv a license to display the preprint in perpetuity. It is made available under aCC-BY 4.0 International license.

Supplementary Figure 2. Phyletic pattern of the presence of the mfp conservon mapped onto a reference phylogeny of the Actinobacteria. Multiple conservons identified for the same genome are separated by a vertical black bar. The scale bar represents the average number of substitutions per site.

bioRxiv preprint doi: https://doi.org/10.64898/2026.03.03.709265; this version posted March 18, 2026. The copyright holder for this preprint (which was not certified by peer review) is the author/funder, who has granted bioRxiv a license to display the preprint in perpetuity. It is made available under aCC-BY 4.0 International license.

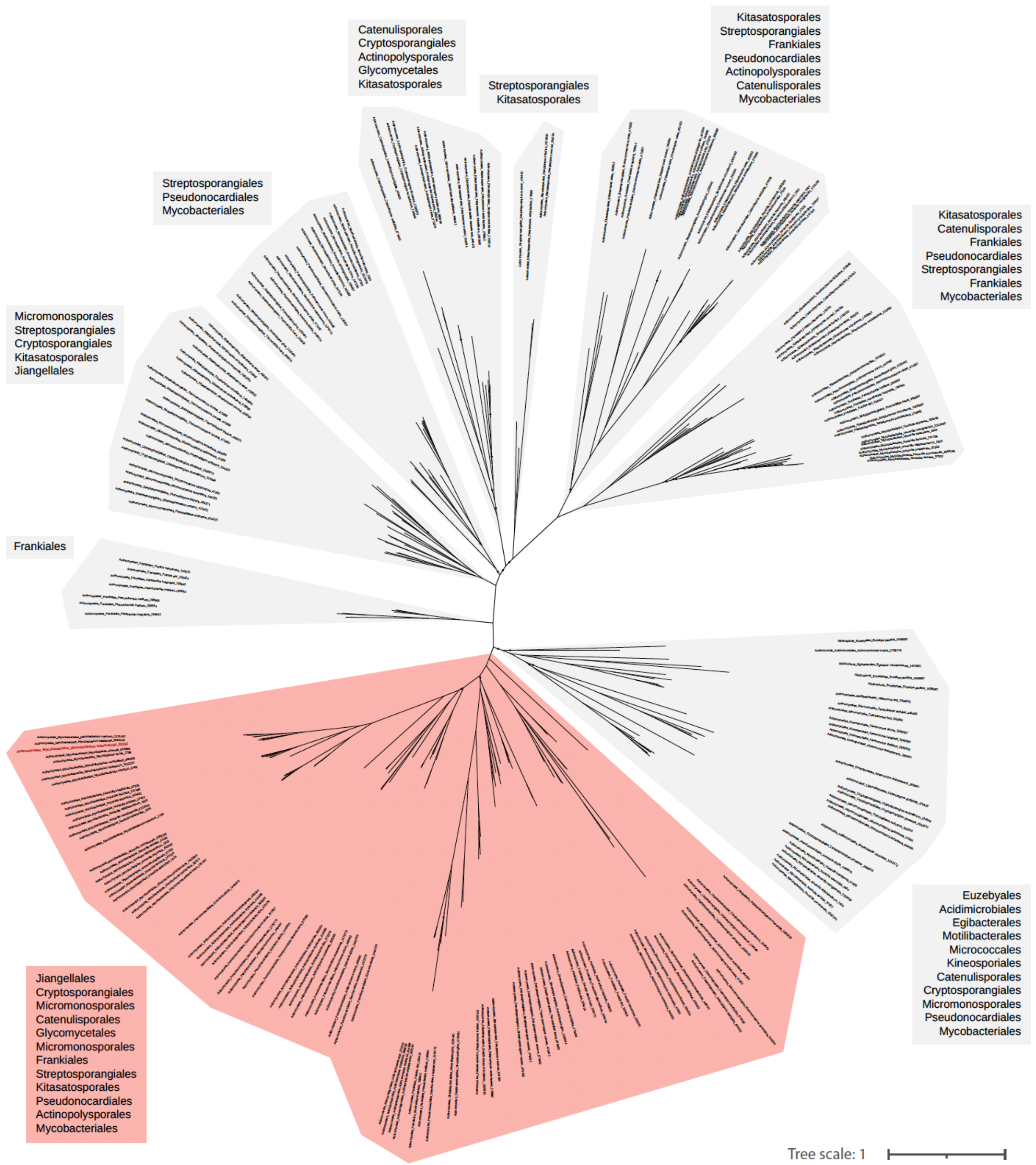

Supplementary Figure 3. Maximum-likelihood phylogeny of the mfp conservon in Actinobacteria. Monophyletic clades are indicated with separate backgrounds. The largest clade is indicated in pink. Note that the topology of this clade roughly matches that of the reference phylogeny of Actinobacteria. UFB <= 80 are indicated with a dot. The scale bar represents the average number of substitutions per site.

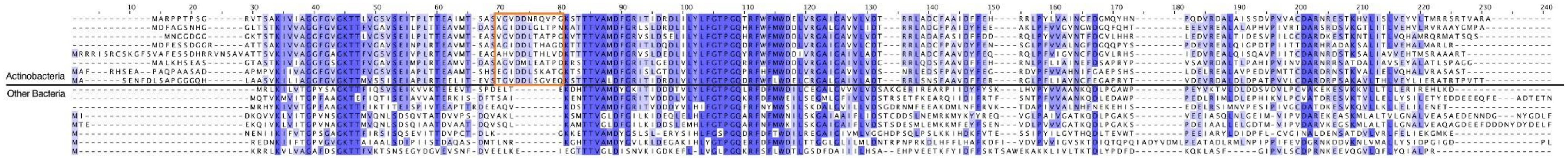

Supplementary Figure 4. Multiple sequence alignment of MfpB including representative sequences of Actinobacteria and other bacterial phyla. The specific actinobacterial insertion sequence is squared in orange.

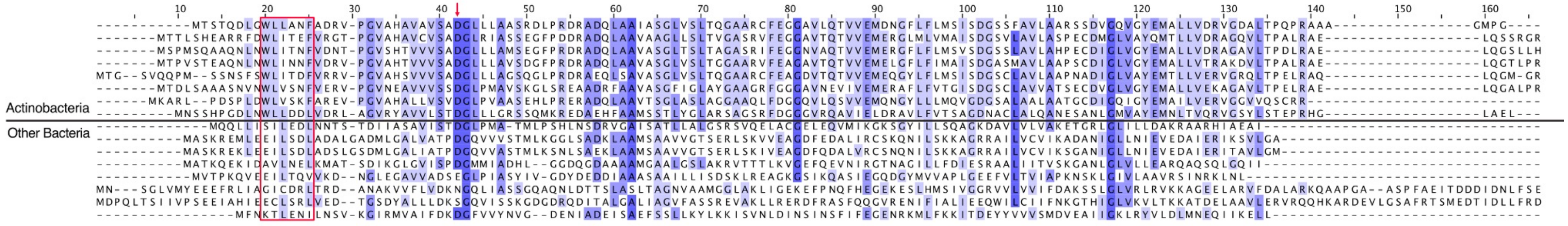

Supplementary Figure 5. Multiple sequence alignment of MfpD including representative sequences of Actinobacteria and other bacterial phyla. The specific actinobacterial sequence ^{12}WLXXXF^{17} (*Mtb* numbering) sequence is squared in red.

bioRxiv preprint doi: https://doi.org/10.64898/2026.03.03.709265; this version posted March 18, 2026. The copyright holder for this preprint (which was not certified by peer review) is the author/funder, who has granted bioRxiv a license to display the preprint in perpetuity. It is made available under aCC-BY 4.0 International license.

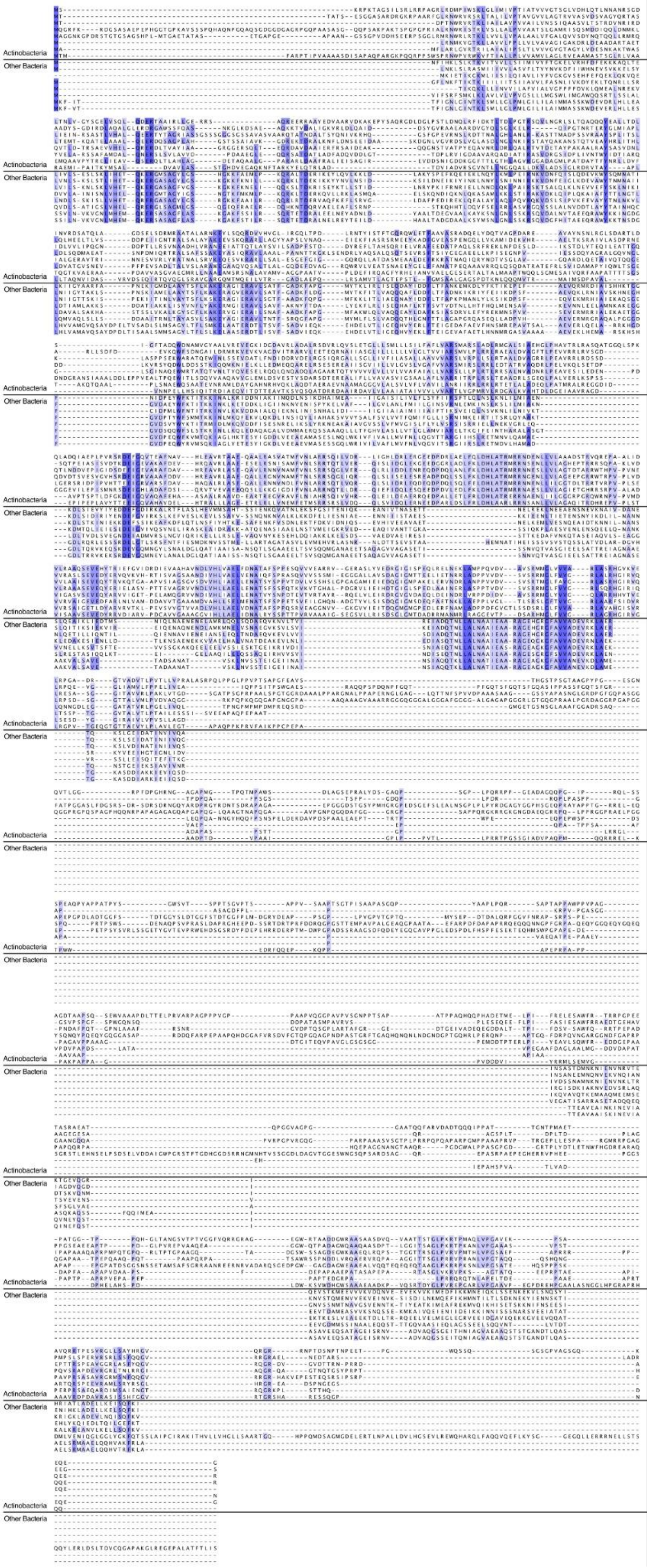

Supplementary Figure 6. Multiple sequence alignment of MfpE including representative sequences of Actinobacteria and other bacterial phyla.

bioRxiv preprint doi: https://doi.org/10.64898/2026.03.03.709265; this version posted March 18, 2026. The copyright holder for this preprint (which was not certified by peer review) is the author/funder, who has granted bioRxiv a license to display the preprint in perpetuity. It is made available under aCC-BY 4.0 International license.

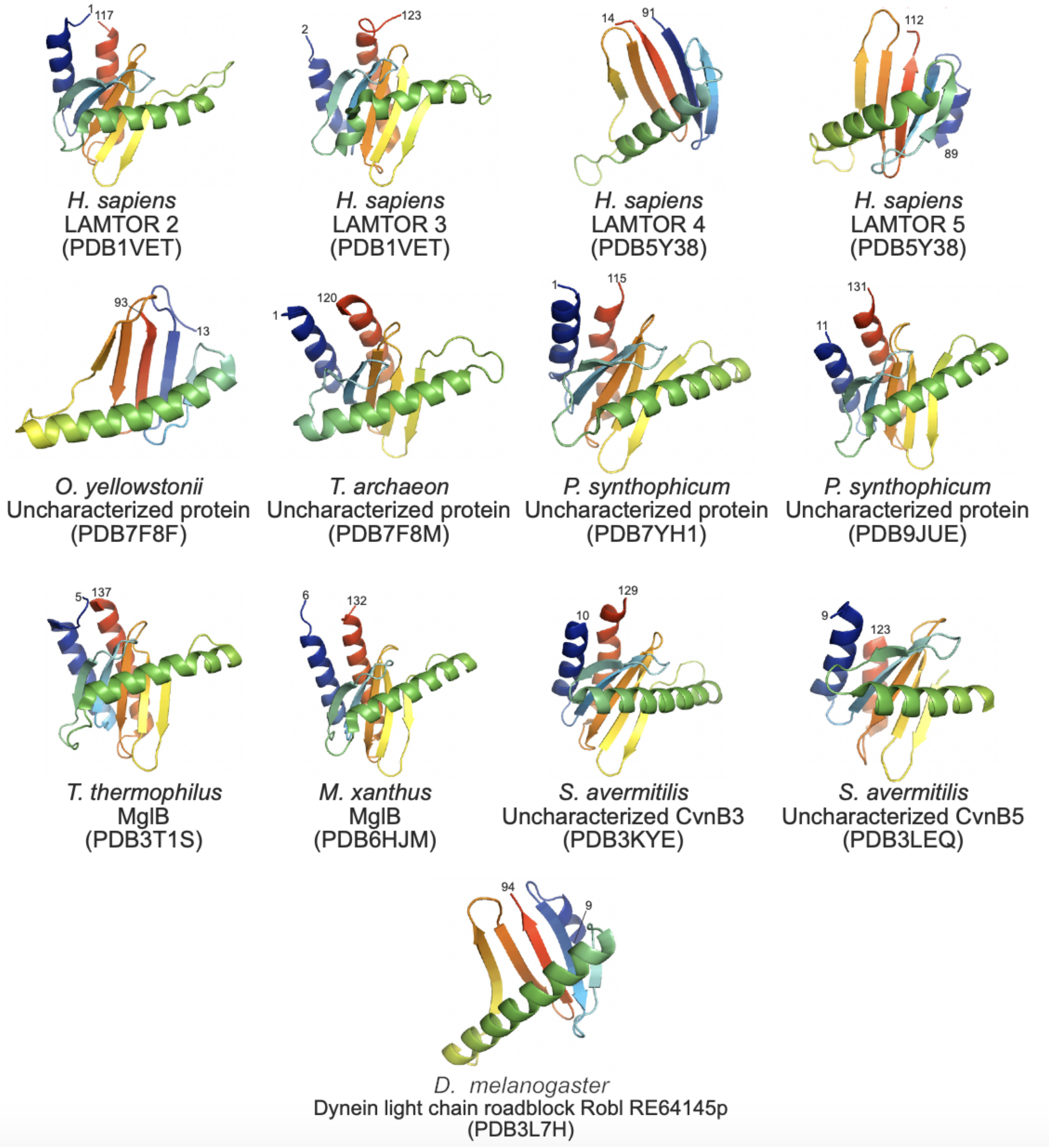

Supplementary Figure 7. Some of the previously solved crystal structures of Roadblock/LC7 domain-containing proteins. Note that all structures have been reported to be in homo- or hetero-dimeric forms.

bioRxiv preprint doi: https://doi.org/10.64898/2026.03.03.709265; this version posted March 18, 2026. The copyright holder for this preprint (which was not certified by peer review) is the author/funder, who has granted bioRxiv a license to display the preprint in perpetuity. It is made available under aCC-BY 4.0 International license.

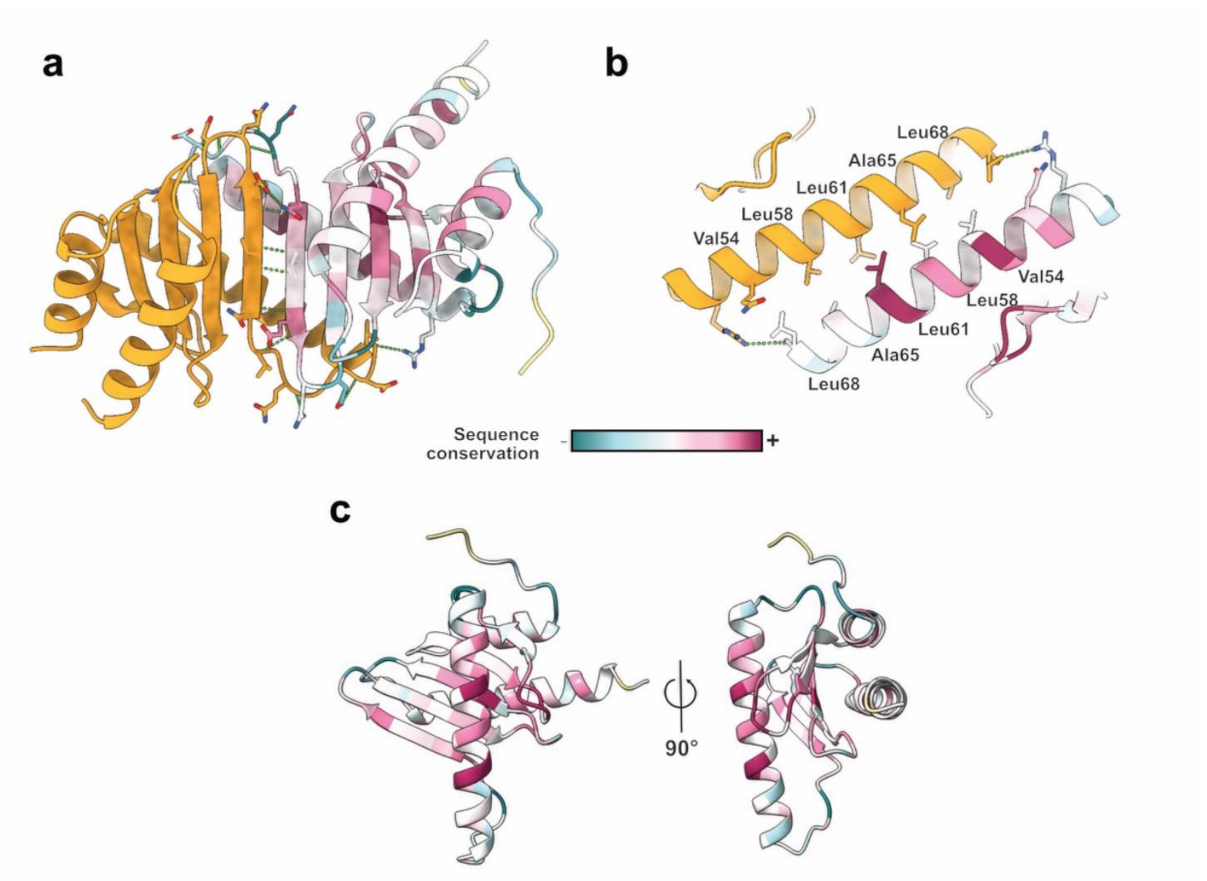

Supplementary Figure 8. Dimerization interface properties of MfpD. One of the two monomers is colored according to a Consurf analysis to highlight conservation patterns in MfpD-related proteins. Hydrogen bonds are represented by green dashed lines, and all polar residues involved in the polar interactions are displayed in stick format.

bioRxiv preprint doi: https://doi.org/10.64898/2026.03.03.709265; this version posted March 18, 2026. The copyright holder for this preprint (which was not certified by peer review) is the author/funder, who has granted bioRxiv a license to display the preprint in perpetuity. It is made available under aCC-BY 4.0 International license.

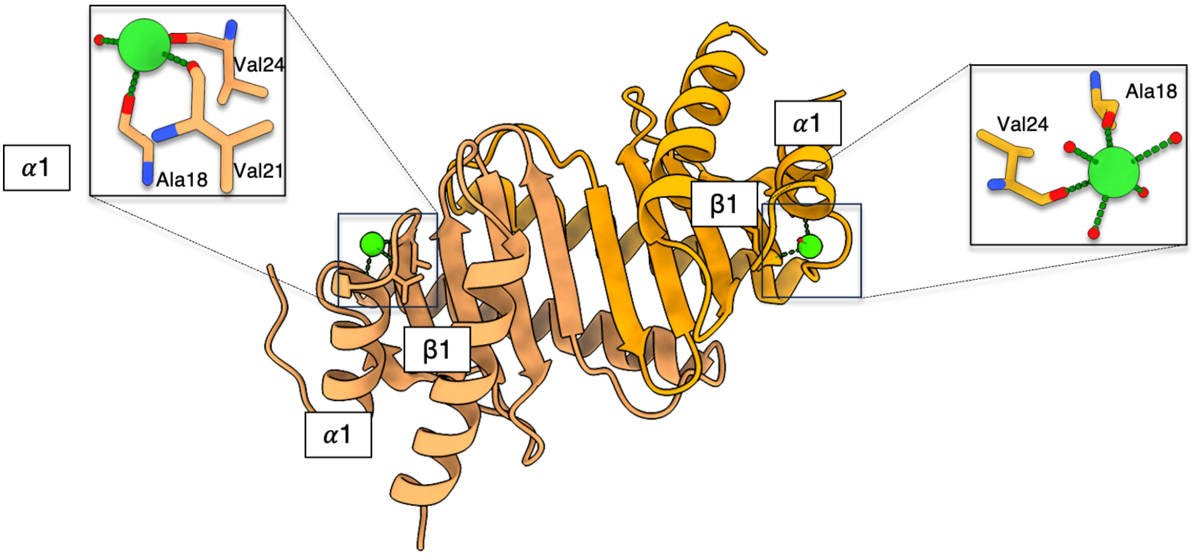

Supplementary Figure 9. Binding of sodium ions within the MfpD dimer.

bioRxiv preprint doi: https://doi.org/10.64898/2026.03.03.709265; this version posted March 18, 2026. The copyright holder for this preprint (which was not certified by peer review) is the author/funder, who has granted bioRxiv a license to display the preprint in perpetuity. It is made available under aCC-BY 4.0 International license.

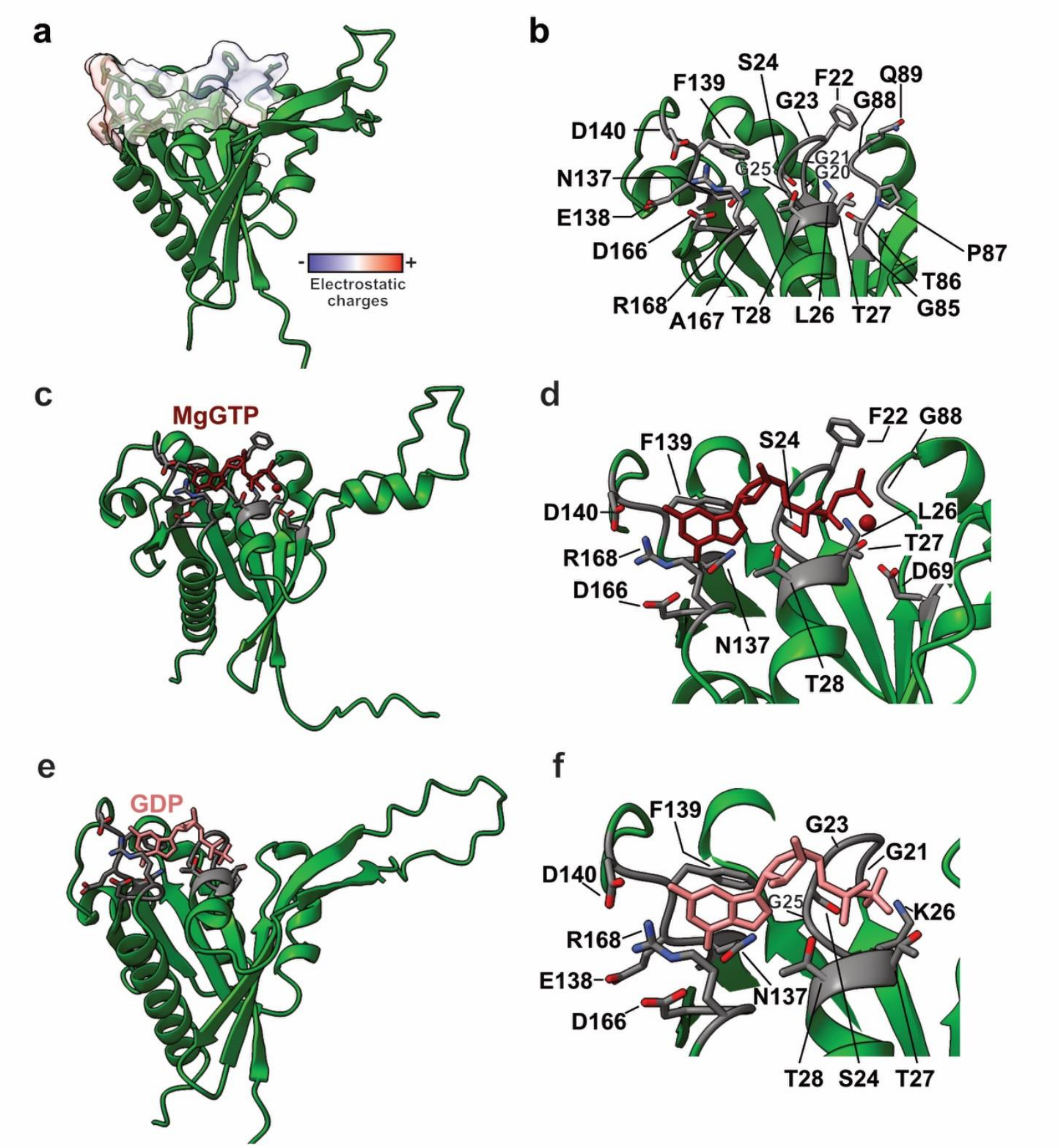

Supplementary Figure 10. AlphaFold-assisted modeling of Mtb MfpB.

AlphaFold3 model of *Mtb* MfpB (***a,b***), *Mtb* MfpB-GTP complex (***c,d***) and *Mtb* MfpB-GDP complex (***e,f***). Zoom on active sites residues in the absence of ligands (***b***) or in the presence of GTP (***d***) or GDP (***f***). The electrostatic charges of the surface of the active site are depicted in ***a***. For simplicity, the unstructured N-terminal sequence is not shown.

bioRxiv preprint doi: https://doi.org/10.64898/2026.03.03.709265; this version posted March 18, 2026. The copyright holder for this preprint (which was not certified by peer review) is the author/funder, who has granted bioRxiv a license to display the preprint in perpetuity. It is made available under aCC-BY 4.0 International license.

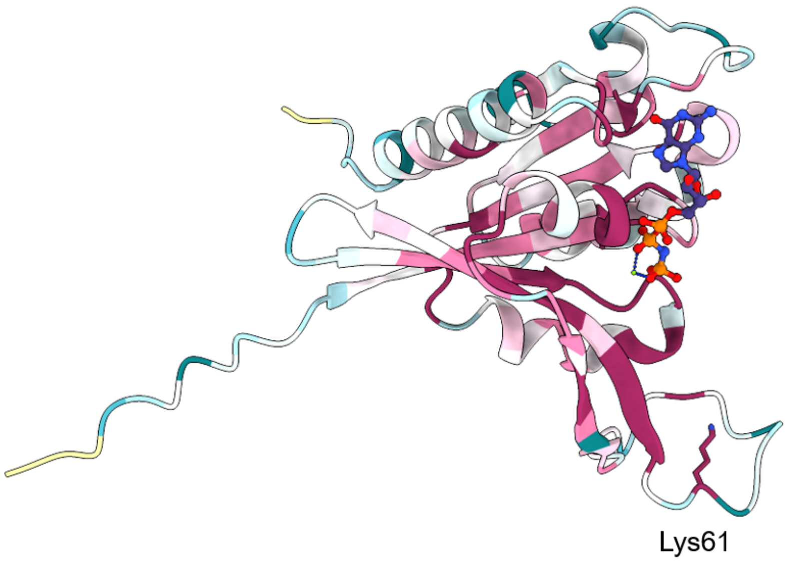

Supplementary Figure 11. Lysine 61 conservation and position in MfpB model

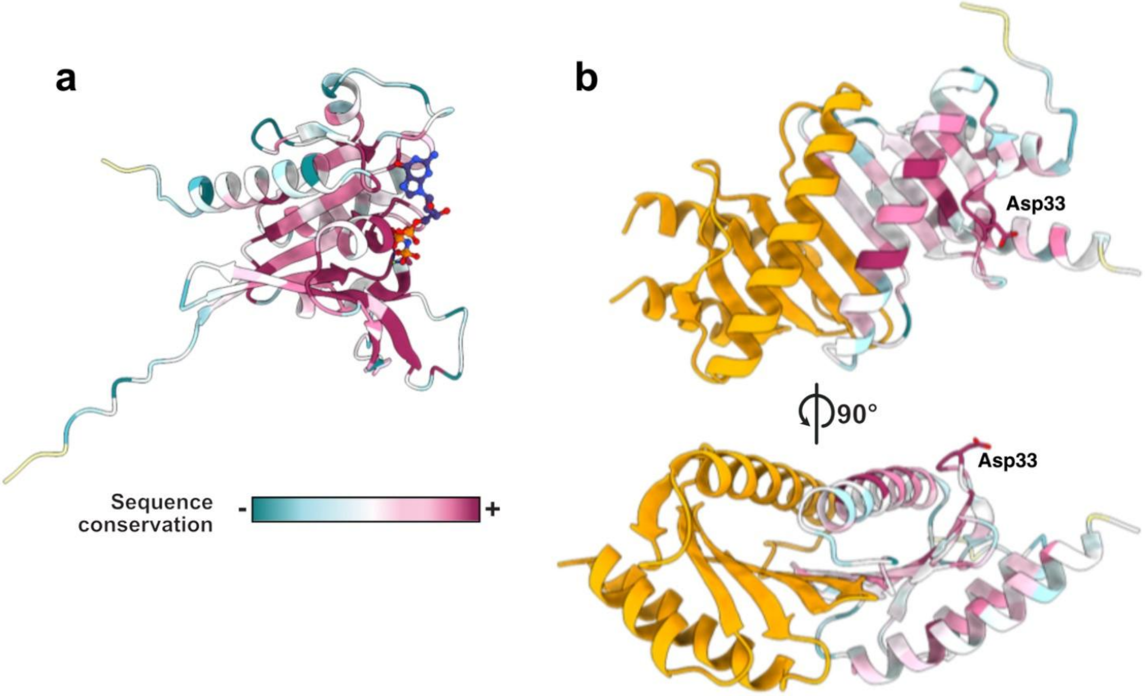

Supplementary Figure 12. Representation of the conservation level of MfpB (a) and aspartate 33 conservation and position in MfpD dimer (b).

bioRxiv preprint doi: https://doi.org/10.64898/2026.03.03.709265; this version posted March 18, 2026. The copyright holder for this preprint (which was not certified by peer review) is the author/funder, who has granted bioRxiv a license to display the preprint in perpetuity. It is made available under aCC-BY 4.0 International license.

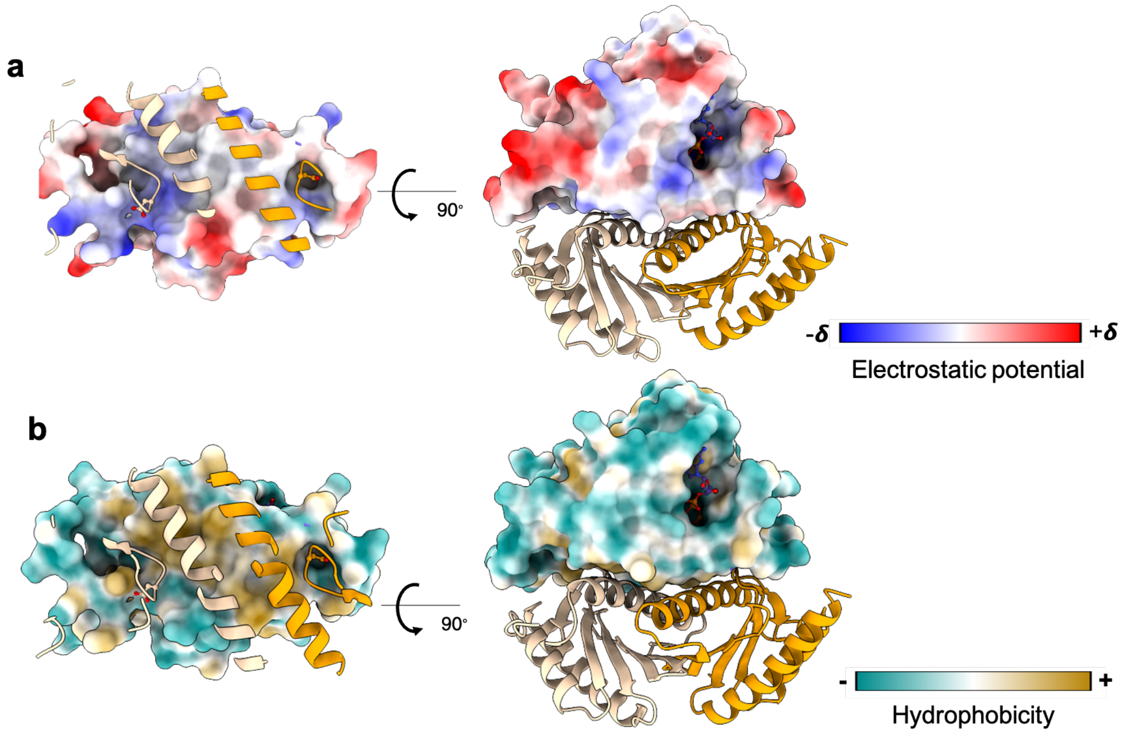

Supplementary Figure 13. 2MfpD-1MfpB complex. a. Electrostatic potential surface and ***b.*** hydrophobicity potential surface for MfpB.

bioRxiv preprint doi: https://doi.org/10.64898/2026.03.03.709265; this version posted March 18, 2026. The copyright holder for this preprint (which was not certified by peer review) is the author/funder, who has granted bioRxiv a license to display the preprint in perpetuity. It is made available under aCC-BY 4.0 International license.

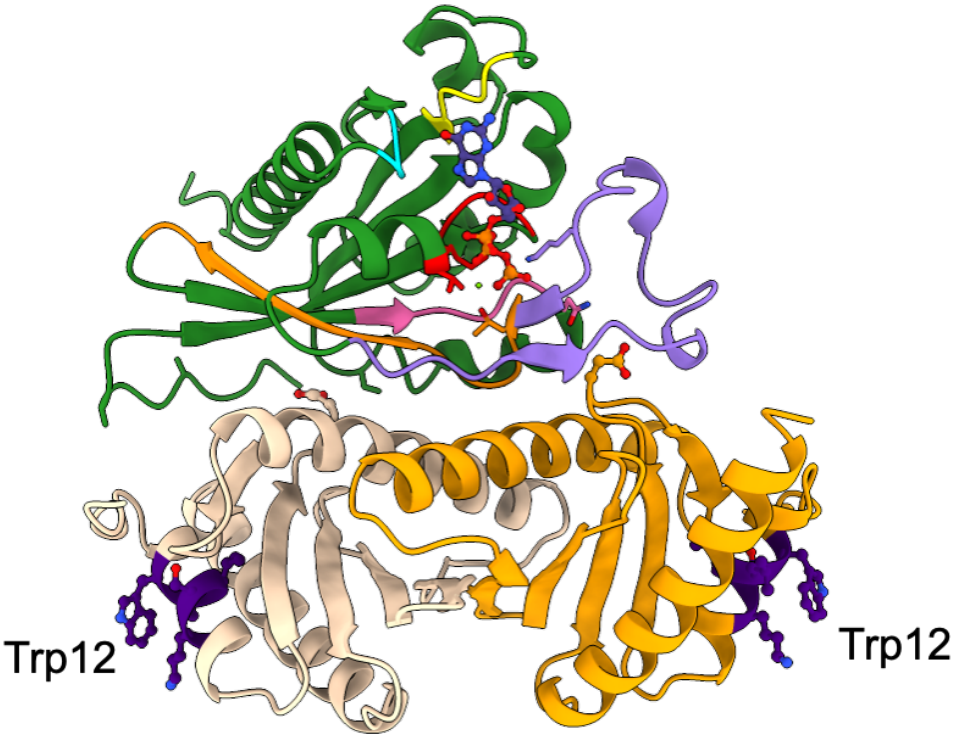

Supplementary Figure 14. Positioning of ^{12}WLVSKF^{17} sequence (in dark purple) in MfpD.

bioRxiv preprint doi: https://doi.org/10.64898/2026.03.03.709265; this version posted March 18, 2026. The copyright holder for this preprint (which was not certified by peer review) is the author/funder, who has granted bioRxiv a license to display the preprint in perpetuity. It is made available under aCC-BY 4.0 International license.

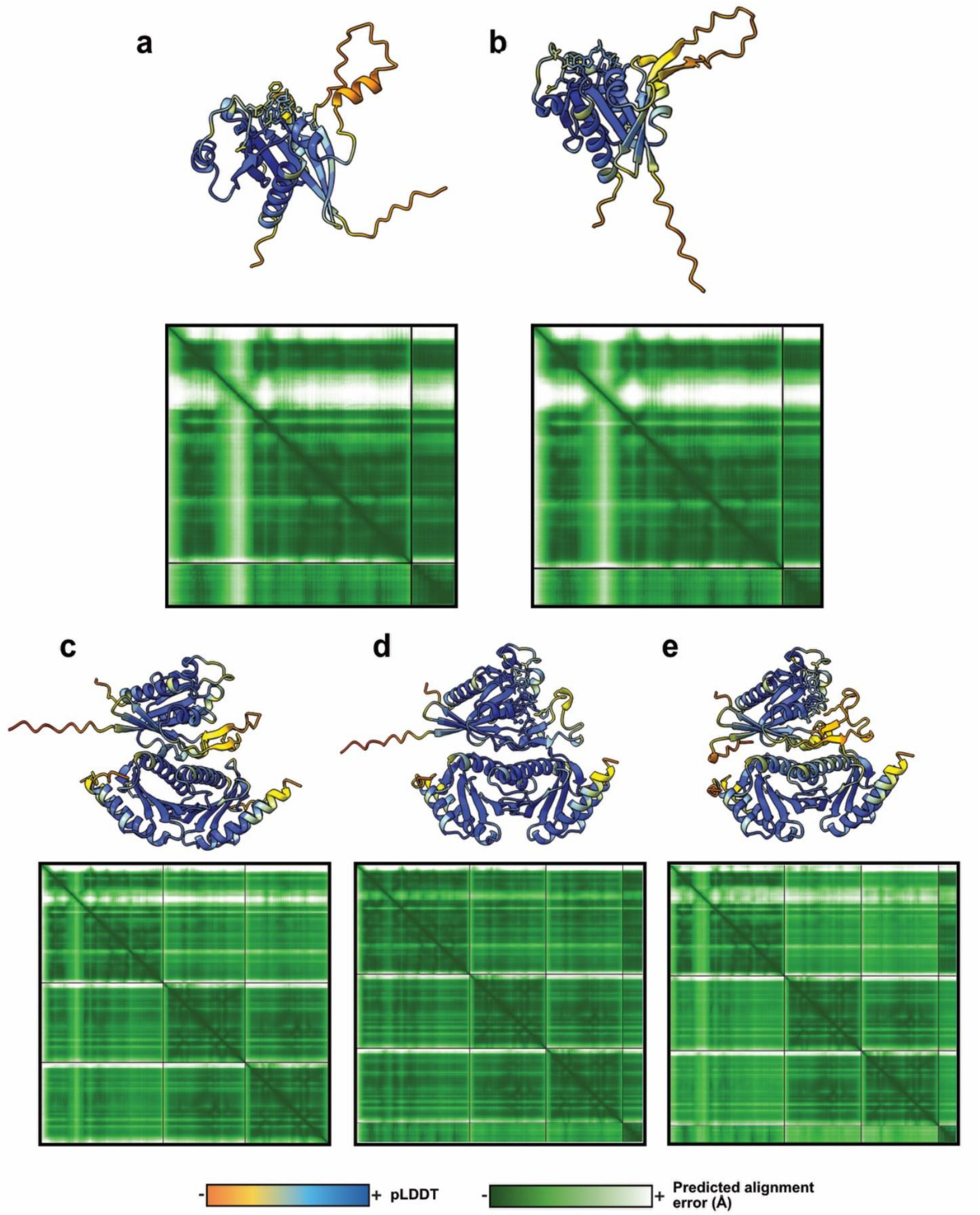

Supplementary Figure 15. AlphaFold3 prediction confidence levels. Three-dimensional structures of MfpB-MgGTP (a), MfpB-GDP (b), MfpD-MfpB (c), MfpD-MfpB-MgGTP and (d) MfpD-MfpB-GDP models colored per-residue confidence level and corresponding residue-residue alignment confidence plots.
